# Origins and Diversification of Myiasis Across Blowflies

**DOI:** 10.1002/ece3.70993

**Published:** 2025-02-13

**Authors:** Gisele Antoniazzi Cardoso, Vanessa A. S. Cunha, Bruno C. Genevcius, Tais Madeira‐Ott, Bárbara Maria de Andrade Costa, Daniela Munhoz Rossoni, Patricia Jacqueline Thyssen, Tatiana Teixeira Torres

**Affiliations:** ^1^ Department of Genetics and Evolutionary Biology Institute of Biosciences, University of São Paulo São Paulo Brazil; ^2^ Department of Zoology Institute of Biosciences, University of São Paulo São Paulo Brazil; ^3^ Department of Animal Biology Institute of Biology, Universidade Estadual de Campinas, UNICAMP São Paulo Brazil

**Keywords:** Diptera, evolutionary transitions, facultative parasitism, trophic habit, vertebrate parasitism

## Abstract

Parasitism represents a prevalent and successful ecological strategy that has evolved independently numerous times across metazoa. Understanding the origin and diversification of parasitism is a central question in evolutionary biology. This study investigated the evolutionary path leading to a specific form of parasitism in blowflies known as myiasis, where larvae develop on or within a vertebrate. We modeled myiasis‐associated traits, including trophic specialization (obligatory parasitism, facultative parasitism and saprophagy), larval food substrate (necrotic, fresh or both) and developmental temperature (constant, variable or both) across the blowfly phylogeny. Our results suggested that the ancestral state of blowflies likely encompassed saprophagy or facultative parasitism, with larvae developing in corpses or necrotic tissues from wounds in either homeothermic or heterothermic hosts. Furthermore, our analysis highlights the role of facultative parasitism as an intermediate step for obligate parasitism in blowflies, indicating that pre‐adaptations for a facultative parasitic lifestyle may serve as stepping stones for emerging obligate parasitism. These findings shed light on the complex evolutionary history of blowfly vertebrate parasitism, emphasizing the importance of facultative parasitism as a critical transitional stage in this evolutionary process.

## Graphical abstract

This study investigates the evolutionary path to parasitism in blowflies, modeling traits such as trophic specialization, larval food substrate, and developmental temperature. Our results support that blowfly parasitism evolved from saprophagy or facultative parasitism, emphasizing the crucial role of facultative parasitism as a transitional stage toward obligate parasitism.

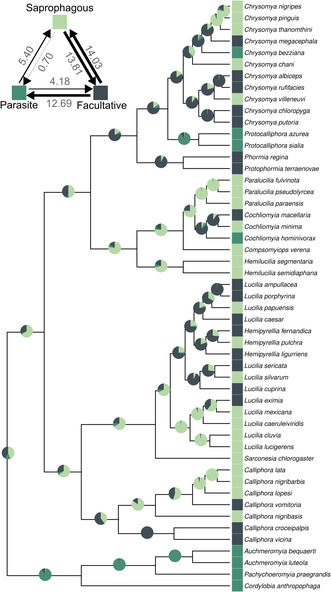

## 1 Introduction

Parasitism is a highly successful and common ecological strategy (Poulin and Morand 2000; Lafferty et al. 2008) that has evolved over 200 times independently across the metazoa (Weinstein and Kuris 2016). Despite its prevalence and ecological importance, our understanding of the tempo and mode of parasite diversification remains limited (Bordes and Morand 2009; Medina et al. 2020). Key questions, such as the evolutionary pace of common parasitic adaptations like genome reduction or attachment apparatuses, and whether the transition to parasitism occurs abruptly through evolutionary leaps or involves intermediate steps, remain unanswered. Addressing these questions requires more macro‐evolutionary studies to reconstruct the evolutionary patterns and rates of parasitic lifestyle traits.

The various independent transitions to parasitism across animal groups provide an opportunity to explore the evolution of the parasitic lifestyle in diverse contexts. Within Diptera, for example, there have been 60 transitions to parasitism (Weinstein and Kuris 2016). Dipterans exhibit a distinct form of parasitism known as myiasis, where larvae develop on or within a vertebrate (Zumpt 1965). This behavior can be obligatory, with larvae feeding exclusively on living tissues (Zumpt 1965; Hall and Wall 1995; Stevens 2003) or facultative, where larvae have the flexibility to initiate myiasis or live as saprophagous (Hall and Wall 1995; Stevens 2003). Saprophagous represents another trophic specialization, where larvae feed on decaying organic matter and may secondarily invade existing infestations to feed on necrotic tissues (Hall and Wall 1995; Stevens 2003). These three trophic specializations are particularly prevalent in blowflies (Calliphoridae family). The existence of a flexible feeding behavior, represented by facultative parasites, presents a unique opportunity to investigate the steps involved in the evolution of parasitism.

Previous studies on parasitism evolution have mainly focused on assigning trophic specializations along branches in phylogenies (with no formal character mapping and testing) (Stevens 2003; Stevens and Wallman 2006; McDonagh and Stevens 2011; Nasser et al. 2021). Since it is hypothesized that parasitism in Calliphoridae evolved progressively, originating from a saprophagous ancestor and passing through a facultative state (Zumpt 1965), formally inferring the origin and transitions of blowfly trophic specialization is crucial for analyzing the evolution of parasitism. Additionally, opportunistic species that initially exploited dead tissues would eventually change to feed on living tissues on living hosts. This transition suggests that tissue type and temperature (environmental temperature, and heterothermic or homeothermic host) may significantly influence the evolutionary pathways of parasitism. Despite the pivotal role of temperature in insect survival and development (Alonso et al. 2015; Yang and Shiao 2014; Grassberger and Reiter 2002; Lecheta et al. 2015; Ratte 1985; Du Plessis et al. 2020; Kotzé et al. 2015; Ma et al. 2021), this variable is often overlooked in parasitism studies. While parasitism in blowflies extends beyond vertebrate parasitism (myiasis) with examples including parasitism on woodlice, earthworms, and termites (Stevens and Wallman 2006; McDonagh and Stevens 2011; Nasser et al. 2021; Marinho et al. 2012), myiasis represents a unique ecological and evolutionary challenge and is the most well‐documented and impactful form of parasitism within the group. Here, we used phylogenetic comparative methods to investigate the route through which myiasis emerged in Calliphoridae. First, we conducted an extensive literature search on distinct myiasis‐associated traits, considering trophic specialization, tissue type, and temperature. Then, we modeled their evolution across the blowfly phylogeny, including 61 species. Our study contributes to a deeper understanding of the mechanism driving parasitism at macroevolutionary scales and its broader implications for evolutionary biology.

## 2 Methods

### 2.1 Myiasis‐Associated Traits

To investigate the origin and evolution of myiasis, we examined three key traits: trophic specialization, larval food substrate, and larval development temperature. We examined these traits separately, as they reflect different physiological adaptations of substrate selection strategies of egg‐laying females and physiological adaptations of larvae.

Trophic specialization reflects a species' breeding environment, the female's role in selecting oviposition sites, and the location where the larvae could be found (e.g., in a wound or cadaver). We categorized species as “obligatory parasite”, “saprophagous” or “facultative parasite” based on their ability to initiate or exploit existing myiasis, following Hall and Wall (1995) and Stevens (2003). Obligatory and facultative parasites can initiate myiasis, acting as primary invaders, with larvae feeding on living tissues. Facultative parasites and saprophagous species can exploit existing infestations with larvae feeding on necrotic tissue, acting as secondary invaders, or exploit corpses and feed on decaying organic matter. We assigned “NA” to species where information on primary or secondary invasion was unavailable in the literature, as this information is crucial for understanding the female's attraction to fresh or necrotic tissue.

Larval food substrate reflects the specific substrate that larvae exploit for nutrition, and it was categorized as “necrotic”, “fresh” or “both”. “Necrotic” refers to decaying organic matter, while “fresh” refers to living tissues on a host. For that, we collected data on the tissue where larvae were found in the field or in the wound, categorizing it as necrotic, fresh, or both. When the specific tissue type was not mentioned in the literature source, we adopted the coding “NA” to indicate that the information was unavailable.

Larval developmental temperature reflects the temperature conditions where larvae develop, based on the temperature where larvae were collected. It was classified as “constant”, “variable” or “both”. “Constant” temperature indicates development in a homeothermic host, while “variable” temperature refers to larvae developing in an environment with fluctuating temperatures, such as a heterothermic host or a cadaver. We used “NA” to indicate that the information where the larvae were collected was not mentioned in the literature.

We conducted literature searches on Google Scholar, Web of Science, and Scopus for each blowfly species name. We refined our searches by adding “myiasis” to specifically identify myiasis cases (i.e., when parasitism affects a living host). This approach enables us to collect data on myiasis‐associated traits for each species (Table 1).

**TABLE 1:** Classification of the blowfly lifestyle into three categorical traits: (i) trophic specialization, (ii) larval food substrate, and (iii) developmental temperature.

Species	Trophic specialization		Larval food substrate		Developmental temperature	
Auchmeromyia bequaerti	Obligatory parasite	(Zumpt 1965)	Fresh	^a^	Constant	(Zumpt 1965)
*Auchmeromyia luteola*	Obligatory parasite	(Stevens 2003)	Fresh	(Garrett‐Jones 1951)	Constant	(Zumpt 1965; McDonagh and Stevens 2011)
*Cordylobia anthropophaga*	Obligatory parasite	(Stevens 2003; Song et al. 2017; Dehecq et al. 2005; Veraldi et al. 1993; Günther 1971; Kouam et al. 2017)	Fresh	(Song et al. 2017; Günther 1971)	Constant	(Zumpt 1965; Stevens 2003; Song et al. 2017; Dehecq et al. 2005; Veraldi et al. 1993; Günther 1971; Kouam et al. 2017; Bernhardt et al. 2019; Kuria and Oyedeji 2020; Sherman 2000)
*Pachychoeromyia praegrandis*	Obligatory parasite	(Zumpt 1965)	Fresh	^a^	Constant	(Zumpt 1965)
*Aldrichina grahami*	NA	NA	Necrotic	(Wang et al. 2018; Li et al. 2011; Yan et al. 2008)	NA	NA
*Calliphora croceipalpis*	Facultative parasite	(Zumpt 1965)	NA	NA	Both	(Zumpt 1965)
*Calliphora lata*	Saprophagous	(Saigusa et al. 2005)	Necrotic	(Saigusa et al. 2005)	Variable	(Saigusa et al. 2005)
*Calliphora lopesi*	Saprophagous	(Gaedke and da Silva Mouga 2017)	Necrotic	(Gaedke and da Silva Mouga 2017)	Variable	(Gaedke and da Silva Mouga 2017)
*Calliphora maestrica*	NA	NA	NA	NA	NA	NA
*Calliphora nigribarbis*	Saprophagous	(Park et al. 2022; Komagata et al. 2006; Centeno et al. 2002)	Necrotic	(Centeno et al. 2002)	Variable	(Park et al. 2022; Komagata et al. 2006; Centeno et al. 2002)
*Calliphora nigribasis*	Saprophagous	(Martinez et al. 2007; Vélez and Wolff 2008)	Necrotic	(Martinez et al. 2007; Vélez and Wolff 2008)	Variable	(Martinez et al. 2007; Vélez and Wolff 2008)
*Calliphora vicina*	Facultative parasite	(Zumpt 1965; Salvetti et al. 2012; Williams et al. 2016; Francesconi and Lupi 2012; Pezzi, Scapoli, Chicca et al. 2021; Pezzi et al. 2017; Scaravelli et al. 2017)	Necrotic	(Saigusa et al. 2005; Centeno et al. 2002; Scaravelli et al. 2017; Amendt et al. 2024; Anderson 1995; Arnaldos et al. 2005; Smeeton et al. 1984; Battán Horenstein et al. 2012; Armani and Dahinten 2017; Rivers et al. 2023; Gemmellaro et al. 2018; Fremdt et al. 2012; Smith and Wall 1997; Davies 1999; Sanford 2017; Alejandra Labud et al. 2003)	Both	(Zumpt 1965; Bernhardt et al. 2019; Sherman 2000; Pezzi, Scapoli, Chicca et al. 2021; Pezzi et al. 2017; Scaravelli et al. 2017; Knotek et al. 2005; Morris and Titchener 1997; Zaidi et al. 2016)
*Calliphora vomitoria*	Facultative parasite	(Zumpt 1965; Morris and Titchener 1997; Wood and Slight 1970; Nandi 2002)	Necrotic	(Amendt et al. 2024; Anderson 1995; Gemmellaro et al. 2018; Smith and Wall 1997; Davies 1999; Sanford 2017; Nandi 2002; Bernhardt et al. 2018; Benecke and Lessig 2001)	Both	(Zumpt 1965; Sherman 2000; Morris and Titchener 1997; Bernhardt et al. 2018)
*Chrysomya albiceps*	Facultative parasite	(Zumpt 1965; Stevens 2003; Francesconi and Lupi 2012; Madeira 2001; Morsy et al. 1991; Ceylan et al. 2019; Fernandes et al. 2009; Ferraz et al. 2011)	Both	(Zumpt 1965; Gaedke and da Silva Mouga 2017; Centeno et al. 2002; Vélez and Wolff 2008; Amendt et al. 2024; Arnaldos et al. 2005; Armani and Dahinten 2017; Vairo et al. 2015; Ramos‐Pastrana et al. 2018; Ellison 1990; Ortloff‐Trautmann et al. 2013; Khater 2021; dos Santos et al. 2024)	Both	(Zumpt 1965; Madeira 2001; Ceylan et al. 2019; Fernandes et al. 2009; Ferraz et al. 2011; Schnur et al. 2009)
*Chrysomya bezziana*	Obligatory parasite	(Stevens 2003; Bambaradeniya et al. 2019; Kumarasinghe et al. 2000; Obanda et al. 2013)	Fresh	(Zumpt 1965; Nandi 2002; Kumarasinghe et al. 2000; Kleine et al. 2014)	constant	(Zumpt 1965; Stevens 2003; Bernhardt et al. 2019; Zaidi et al. 2016; Bambaradeniya et al. 2019; Kumarasinghe et al. 2000; Obanda et al. 2013; Kleine et al. 2014; Mondal et al. 2021; Sontigun, Sanit et al. 2018; Sontigun, Sukontason et al. 2018)
*Chrysomya chani*	Saprophagous	(Sukontason et al. 2003; Sukontason et al. 2018; Silahuddin et al. 2015; Sukontason et al. 2007)	Necrotic	(Sukontason et al. 2003; Sukontason et al. 2018; Silahuddin et al. 2015)	Variable	(Sukontason et al. 2003; Sukontason et al. 2018; Silahuddin et al. 2015; Sukontason et al. 2007)
*Chrysomya chloropyga*	Facultative parasite	(Zumpt 1965; Kuria et al. 2015)	Necrotic	(Centeno et al. 2002)	Both	(Zumpt 1965; Kuria et al. 2015)
*Chrysomya marginalis*	NA	NA	Necrotic	(Smeeton et al. 1984; Ellison 1990; Mukandiwa et al. 2012)	Both	(Zumpt 1965)
*Chrysomya megacephala*	Facultative parasite	(Zumpt 1965; Fernandes et al. 2009; Bambaradeniya et al. 2019; Kumarasinghe et al. 2000; Kuria et al. 2015; Sangmala et al. 2020; Senapathi et al. 2023)	Necrotic	(Yan et al. 2008; Gaedke and da Silva Mouga 2017; Centeno et al. 2002; Amendt et al. 2024; Sanford 2017; Nandi 2002; Sukontason et al. 2018; Silahuddin et al. 2015; Sukontason et al. 2007; Nelder et al. 2008; Moophayak et al. 2017; Sukontason, Narongchai, Sripakdee et al. 2005; Sukontason, Narongchai, Sukontason et al. 2005; Nurita and Hassan 2013; Kumara et al. 2012; Ahmad et al. 2020; Chin et al. 2008; Swiger et al. 2014)	Both	(Zumpt 1965; Bernhardt et al. 2019; Kuria and Oyedeji 2020; Zaidi et al. 2016; Fernandes et al. 2009; Bambaradeniya et al. 2019; Kumarasinghe et al. 2000; Mondal et al. 2021; Kuria et al. 2015; Sangmala et al. 2020; Senapathi et al. 2023; Sukontason, Narongchai, Sripakdee et al. 2005; Sukontason, Narongchai, Sukontason et al. 2005; Azevedo et al. 2015)
*Chrysomya nigripes*	Saprophagous	(Silahuddin et al. 2015; Sukontason et al. 2007)	Necrotic	^b^	Variable	(Silahuddin et al. 2015; Sukontason et al. 2007)
*Chrysomya pinguis*	Saprophagous	(Saigusa et al. 2005; Nandi 2002; Silahuddin et al. 2015; Sukontason et al. 2022; Monum et al. 2017)	Necrotic	(Saigusa et al. 2005; Nandi 2002; Silahuddin et al. 2015; Sukontason et al. 2022; Monum et al. 2017)	variable	(Saigusa et al. 2005; Nandi 2002; Silahuddin et al. 2015; Sukontason et al. 2022; Monum et al. 2017)
*Chrysomya putoria*	Facultative parasite	(Zumpt 1965)	Necrotic	(Amendt et al. 2024)	Both	(Zumpt 1965; Otesile and Dipeolu 1981)
*Chrysomya rufifacies*	Facultative parasite	(Zumpt 1965; Stevens 2003; Francesconi and Lupi 2012; Nandi 2002; Baumgartner 1993; Sanford et al. 2014)	Both	(Smeeton et al. 1984; Sanford 2017; Nandi 2002; Silahuddin et al. 2015; Sukontason et al. 2007; Nelder et al. 2008; Moophayak et al. 2017; Sukontason, Narongchai, Sripakdee et al. 2005; Sukontason, Narongchai, Sukontason et al. 2005; Kumara et al. 2012; Ahmad et al. 2020; Chin et al. 2008; Swiger et al. 2014; Baumgartner 1993; Sanford et al. 2014; Bambaradeniya et al. 2021; Barrett et al. 2022)	Both	(Zumpt 1965; Bernhardt et al. 2019; Zaidi et al. 2016; Baumgartner 1993; Sanford et al. 2014; Bambaradeniya et al. 2021; Bermúdez et al. 2007)
*Chrysomya thanomthini*	Saprophagous	(Silahuddin et al. 2015)	Necrotic	^b^	Variable	(Silahuddin et al. 2015)
*Chrysomya villeneuvi*	Saprophagous	(Sukontason et al. 2003; Sukontason et al. 2018; Silahuddin et al. 2015; Sukontason et al. 2007; Kumara et al. 2012; Ahmad et al. 2020; Monum et al. 2017; Sukontason, Sukontason, Piangjai et al. 2005)	Necrotic	(Sukontason et al. 2003; Sukontason et al. 2018; Silahuddin et al. 2015; Kumara et al. 2012; Ahmad et al. 2020; Monum et al. 2017)	Variable	(Sukontason et al. 2003; Sukontason et al. 2018; Silahuddin et al. 2015; Sukontason et al. 2007; Kumara et al. 2012; Ahmad et al. 2020; Monum et al. 2017; Sukontason, Sukontason, Piangjai et al. 2005)
*Cochliomyia aldrichi*	NA	NA	NA	NA	NA	NA
*Cochliomyia hominivorax*	Obligatory parasite	(Stevens 2003; Madeira 2001; Fernandes et al. 2009; Cansi and Demo 2011; Calvopina et al. 2020)	Fresh	(Trombetta et al. 2009; Durão et al. 2017)	Constant	(Stevens 2003; Bernhardt et al. 2019; Sherman 2000; Madeira 2001; Fernandes et al. 2009; Ferraz et al. 2011; Azevedo et al. 2015; Cansi and Demo 2011; Calvopina et al. 2020; Trombetta et al. 2009; Durão et al. 2017; Thyssen et al. 2012)
*Cochliomyia macellaria*	Facultative parasite	(Ferraz et al. 2011; Chodosh et al. 1991; Harrison and Pearson 1968; Farias et al. 2020)	Necrotic	(Centeno et al. 2002; Vélez and Wolff 2008; Amendt et al. 2024; Battán Horenstein et al. 2012; Sanford 2017; Alejandra Labud et al. 2003; Ramos‐Pastrana et al. 2018; Ortloff‐Trautmann et al. 2013; Nelder et al. 2008; Swiger et al. 2014; Barrett et al. 2022; Harrison and Pearson 1968; Boatright and Tomberlin 2010; Battán Horenstein et al. 2023)	Both	(Sherman 2000; Ferraz et al. 2011; Bermúdez et al. 2007; Chodosh et al. 1991; Harrison and Pearson 1968; Farias et al. 2020)
*Cochliomyia minima*	Saprophagous	(Yusseff‐Vanegas 2014; Vergara‐Pineda et al. 2012)	Necrotic	(Yusseff‐Vanegas 2014; Vergara‐Pineda et al. 2012)	Variable	(Yusseff‐Vanegas 2014; Vergara‐Pineda et al. 2012)
*Compsomyiops fulvicrura*	NA	NA	NA	NA	NA	NA
*Compsomyiops verena*	Saprophagous	(Martinez et al. 2007)	Necrotic	(Martinez et al. 2007)	Variable	(Martinez et al. 2007)
*Hemilucilia segmentaria*	Saprophagous	(Gaedke and da Silva Mouga 2017; Amendt et al. 2024)	Necrotic	(Gaedke and da Silva Mouga 2017; Amendt et al. 2024)	Variable	(Gaedke and da Silva Mouga 2017; Amendt et al. 2024)
*Hemilucilia semidiaphana*	Saprophagous	(Gaedke and da Silva Mouga 2017; Amendt et al. 2024; Vairo et al. 2015; Ramos‐Pastrana et al. 2018; Moura et al. 1997)	Necrotic	(Gaedke and da Silva Mouga 2017; Amendt et al. 2024; Vairo et al. 2015; Ramos‐Pastrana et al. 2018; Moura et al. 1997)	Variable	(Gaedke and da Silva Mouga 2017; Amendt et al. 2024; Vairo et al. 2015; Ramos‐Pastrana et al. 2018; Moura et al. 1997)
*Paralucilia fulvinota*	Saprophagous	(Amendt et al. 2024)	Necrotic	(Amendt et al. 2024)	Variable	(Amendt et al. 2024)
*Paralucilia paraensis*	Saprophagous	(Sales et al. 2013)	Necrotic	(Sales et al. 2013)	Variable	(Sales et al. 2013)
*Paralucilia pseudolyrcea*	Saprophagous	(Centeno et al. 2002)	Necrotic	(Centeno et al. 2002)	Variable	(Centeno et al. 2002)
*Phormia regina*	Facultative parasite	(Matyukhin and Krivosheina 2008)	Necrotic	(Francesconi and Lupi 2012; Amendt et al. 2024; Anderson 1995; Sanford 2017; Bernhardt et al. 2018; Nelder et al. 2008; Swiger et al. 2014; Vergara‐Pineda et al. 2012; Ali‐Khan and Ali‐Khan 1975; Haddow and Thomson 1937)	Both	(Kuria and Oyedeji 2020; Sherman 2000; Bernhardt et al. 2018; Ali‐Khan and Ali‐Khan 1975; Haddow and Thomson 1937; Abdel‐Hafeez et al. 2015; Stewart 1929; Hall et al. 1986; Alexis and Mittleman 1988; Altintop et al. 2022)
*Protocalliphora azurea*	Obligatory parasite	(Zumpt 1965; Stevens 2003; Matyukhin and Krivosheina 2008; Moreno‐Rueda 2021; Hori et al. 1990; Puchala 2004; Belskii et al. 2005; Potti 2008; Krivosheina et al. 2018)	Fresh	(Francesconi and Lupi 2012; Amendt et al. 2024; Anderson 1995; Bernhardt et al. 2018; Moophayak et al. 2017; Vergara‐Pineda et al. 2012; Ali‐Khan and Ali‐Khan 1975; Byrd and Allen 2001)	Constant	(Zumpt 1965; Stevens 2003; Matyukhin and Krivosheina 2008; Moreno‐Rueda 2021; Hori et al. 1990; Puchala 2004; Belskii et al. 2005; Potti 2008; Krivosheina et al. 2018; Eeva et al. 2015; Aguilar et al. 2016; Castaño‐Vázquez et al. 2021; Garrido‐Bautista et al. 2022)
*Protocalliphora sialia*	Obligatory parasite	(Stevens 2003; Wittmann and Beason 1992; Germaine and Germaine 2002; DeSimone et al. 2018; Grab et al. 2019; Sun et al. 2020; Knutie 2020; Ingala et al. 2021; Albert et al. 2023)	Fresh	(DeSimone et al. 2018; Williams and Dittmar 2020)	Constant	(Stevens 2003; Wittmann and Beason 1992; Germaine and Germaine 2002; DeSimone et al. 2018; Grab et al. 2019; Sun et al. 2020; Knutie 2020; Ingala et al. 2021; Albert et al. 2023; Williams and Dittmar 2020; Proudfoot et al. 2005; Daoust et al. 2012)
*Protophormia terraenovae*	Facultative parasite	(Zumpt 1965; Morris and Titchener 1997)	Both	(Francesconi and Lupi 2012; Amendt et al. 2024; Anderson 1995; Davies 1999; Bernhardt et al. 2018; Ourrad et al. 2022)	Both	(Zumpt 1965; Morris and Titchener 1997; Kleine et al. 2014; Haddow and Thomson 1937; Ourrad et al. 2022)
*Hemipyrellia fernandica*	Facultative parasite	(Jong and Krell 2011)	Necrotic	(Jong and Krell 2011)	Both	(Jong and Krell 2011)
*Hemipyrellia ligurriens*	Facultative parasite	(Williams et al. 2016; Sinha 2012)	Necrotic	(Silahuddin et al. 2015; Nurita and Hassan 2013; Kumara et al. 2012; Ahmad et al. 2020; Eliza and Zuha 2018)	Both	(Zaidi et al. 2016; Sinha 2012)
*Hemipyrellia pulchra*	Saprophagous	(Williams et al. 2016; Nandi 2002)	Necrotic	(Nandi 2002)	Variable	(Williams et al. 2016; Nandi 2002)
*Lucilia ampullacea*	Facultative parasite	(Zumpt 1965; Williams et al. 2016; Glaw et al. 2014)	Necrotic	(Amendt et al. 2024; Fremdt et al. 2012; Smith and Wall 1997; Nandi 2002; Glaw et al. 2014)	Both	(Zumpt 1965; Glaw et al. 2014)
*Lucilia caesar*	Facultative parasite	(Zumpt 1965; Stevens 2003; Williams et al. 2016; Morris and Titchener 1997; Haddow and Thomson 1937; Pezzi et al. 2021b; Gao et al. 2021; Franza et al. 2006)	Necrotic	(Amendt et al. 2024; Smith and Wall 1997; Davies 1999; Bernhardt et al. 2018; Martínez‐Calabuig et al. 2021)	Both	(Zumpt 1965; Bernhardt et al. 2019; Morris and Titchener 1997; Bernhardt et al. 2018; Haddow and Thomson 1937; Pezzi, Scapoli, Wyatt, and Bonacci, 2021; Gao et al. 2021; Franza et al. 2006; Martínez‐Calabuig et al. 2021)
*Lucilia cluvia*	Saprophagous	(Centeno et al. 2002; Williams et al. 2016)	Necrotic	(Centeno et al. 2002)	Variable	(Centeno et al. 2002; Williams et al. 2016)
*Lucilia caeruleiviridis*	Saprophagous	(Nelder et al. 2008; Swiger et al. 2014; Joy et al. 2006)	Necrotic	(Nelder et al. 2008; Swiger et al. 2014; Joy et al. 2006)	Variable	(Nelder et al. 2008; Swiger et al. 2014; Joy et al. 2006)
*Lucilia cuprina*	Facultative parasite	(Zumpt 1965; Stevens 2003; Sherman 2000; Williams et al. 2016; Fernandes et al. 2009; Kuria et al. 2015; Ahadizadeh et al. 2015; Quesada‐Lobo et al. 2012; Kuria et al. 2010; Moradi‐Asl et al. 2021; Lukin 1989; Nazni et al. 2011)	Both	(Zumpt 1965; Yan et al. 2008; Saigusa et al. 2005; Gaedke and da Silva Mouga 2017; Amendt et al. 2024; Sanford 2017; Nandi 2002; Sukontason et al. 2007; Mukandiwa et al. 2012; Nurita and Hassan 2013; Kuria et al. 2010; Tellam and Bowles 1997)	Both	(Zumpt 1965; Bernhardt et al. 2019; Kuria and Oyedeji 2020; Sherman 2000; Zaidi et al. 2016; Fernandes et al. 2009; Kuria et al. 2015; Azevedo et al. 2015; Otesile and Dipeolu 1981; Ahadizadeh et al. 2015; Quesada‐Lobo et al. 2012; Kuria et al. 2010; Moradi‐Asl et al. 2021; Lukin 1989; Nazni et al. 2011)
*Lucilia eximia*	Facultative parasite	(Otesile and Dipeolu 1981; Cansi and Demo 2011; Calvopina et al. 2020; Azeredo‐Espin and Madeira 1996; Moretti and Thyssen 2006)	Both	(Gaedke and da Silva Mouga 2017; Vélez and Wolff 2008; Amendt et al. 2024; Sanford 2017; Sanford et al. 2014; Moura et al. 1997; Azeredo‐Espin and Madeira 1996; Moretti and Thyssen 2006)	Both	(Sanford et al. 2014; Cansi and Demo 2011; Calvopina et al. 2020; Azeredo‐Espin and Madeira 1996; Moretti and Thyssen 2006)
*Lucilia fayeae*	NA	NA	NA	NA	NA	NA
*Lucilia lucigerens*	Saprophagous	(Barrett et al. 2022)	Necrotic	(Barrett et al. 2022)	Variable	(Barrett et al. 2022)
*Lucilia mexicana*	Saprophagous	(Williams et al. 2016; Hall and Townsend 1977)	Necrotic	^b^	Variable	(Williams et al. 2016; Hall and Townsend 1977)
*Lucilia papuensis*	Saprophagous	(Williams et al. 2016)	Necrotic	^b^	Variable	(Williams et al. 2016)
*Lucilia porphyrina*	Facultative parasite	(Zumpt 1965; Williams et al. 2016)	Both	(Zumpt 1965; Nandi 2002; Silahuddin et al. 2015; Monum et al. 2017)	Both	(Zumpt 1965; Zaidi et al. 2016)
*Lucilia retroversa*	NA	NA	NA	NA	NA	NA
*Lucilia rica*	NA	NA	NA	NA	NA	NA
*Lucilia richardsi*	NA	NA	Necrotic	(Davies 1999)	Both	(Zumpt 1965)
*Lucilia sericata*	Facultative parasite	(Zumpt 1965; Stevens 2003; Sherman 2000; Williams et al. 2016; Pezzi, Scapoli, Chicca et al. 2021; Morris and Titchener 1997; Kuria et al. 2015; Haddow and Thomson 1937; Vanin et al. 2017)	Both	(Yan et al. 2008; Centeno et al. 2002; Amendt et al. 2024; Anderson 1995; Arnaldos et al. 2005; Smeeton et al. 1984; Armani and Dahinten 2017; Smith and Wall 1997; Davies 1999; Sanford 2017; Alejandra Labud et al. 2003; Bernhardt et al. 2018; Vairo et al. 2015; Ortloff‐Trautmann et al. 2013; Swiger et al. 2014; Battán Horenstein et al. 2023; Vergara‐Pineda et al. 2012; Ourrad et al. 2022; Vanin et al. 2017; Baumjohann et al. 2011; De Jong 2014)	Both	(Zumpt 1965; Bernhardt et al. 2019; Sherman 2000; Pezzi, Scapoli, Chicca et al. 2021; Pezzi et al. 2017; Morris and Titchener 1997; Zaidi et al. 2016; Bernhardt et al. 2018; Schnur et al. 2009; Kuria et al. 2015; Haddow and Thomson 1937; Ourrad et al. 2022; Vanin et al. 2017; Anderson and Huitson 2004)
*Lucilia silvarum*	Saprophagous	(Burgess 2022)	Necrotic	(Fremdt et al. 2012)	Both	(Burgess 2022)
*Sarconesia chlorogaster*	Saprophagous	(Centeno et al. 2002; Alejandra Labud et al. 2003; Vairo et al. 2015; Battán Horenstein et al. 2023; Moura et al. 1997; Castillo et al. 2017)	Necrotic	(Centeno et al. 2002; Alejandra Labud et al. 2003; Vairo et al. 2015; Battán Horenstein et al. 2023; Moura et al. 1997; Castillo et al. 2017)	Variable	(Centeno et al. 2002; Alejandra Labud et al. 2003; Vairo et al. 2015; Battán Horenstein et al. 2023; Moura et al. 1997; Castillo et al. 2017)
*Sarconesia versicolor*	NA	NA	NA	NA	NA	NA

^a^ We considered that obligatory parasitic larvae feed on fresh tissues (No reference about what larvae eat and no forensic case or larvae on cadaver was found for these species).

^b^ We considered that saprophagous larvae feed on necrotic tissues (No reference about what larvae eat and no myiasis case was found for these species).

Abbreviation: NA, not available.

While other forms of parasitic lifestyle, such as parasitism on woodlice, earthworms, and termites (Stevens and Wallman 2006; McDonagh and Stevens 2011; Nasser et al. 2021; Marinho et al. 2012), are present in blowflies, they are not covered in this study due to the lack of information about them in the literature and our focus on vertebrate parasitism (myiasis).

### 2.2 Phylogenetic Inference

While existing blowfly phylogenies are available (Stevens 2003; Stevens and Wallman 2006; McDonagh and Stevens 2011; Marinho et al. 2012; Yan et al. 2021; Wallman et al. 2005; Kutty et al. 2019; Cerretti et al. 2017), there is a need for a comprehensive one covering a diverse range of species. We built a phylogeny comprising 61 calliphorid species and three Sarcophagidae species (*Oxysarcodexia thornax*, *Sarcophaga bullata* and *Peckia ingens*) as outgroups (Table 2). We used markers from both the mitochondrial genome (COI and the 3′ portion 16S rDNA) and the nuclear genome (ITS2 and the 5′ region of the 28S rDNA).

**TABLE 2:** Species used in the phylogenetic inference and their GenBank accession numbers.

Family	Species	GenBank accession number			
		COI	ITS2	16S	28S
Calliphoridae	*Auchmeromyia bequaerti* (Roubaud, 1913)	—	JQ246578	—	JQ246626
Calliphoridae	*Auchmeromyia luteola* (Fabricius, 1805)	FR719153	—	—	AJ551431
Calliphoridae	*Aldrichina grahami* (Aldrich, 1930)	KC354374	KR105493	GQ396703	MF281687
Calliphoridae	*Calliphora croceipalpis* (Jaennicke, 1867)	JQ246671	JQ246573	JQ246720	JQ246616
Calliphoridae	*Calliphora lata* Coquillett, 1898	EU880187	KR105516	—	—
Calliphoridae	*Calliphora lopesi* Mello, 1962	KJ195705	KJ438995	—	—
Calliphoridae	*Calliphora maestrica* Peris, Gonzalez‐Mora, Fernandez and Peris, 1998	MF097186	MF097581	—	—
Calliphoridae	*Calliphora nigribarbis* Snellen van Vollenhoven, 1863	KY001863	—	AB465966	AB466112
Calliphoridae	*Calliphora nigribasis* Macquart, 1851	MG700300	KP723303	—	—
Calliphoridae	*Calliphora vicina* Robineau‐Desvoidy, 1830	JQ246672	EF560178	JQ246720	JQ246617
Calliphoridae	*Calliphora vomitoria* (Linnaeus, 1758)	JQ246673	EF560179	JQ246721	JQ246618
Calliphoridae	*Chrysomya albiceps* (Wiedemann, 1819)	JQ246659	EF560173	JQ246709	JQ246604
Calliphoridae	*Chrysomya bezziana* (Villeneuve, 1914)	JQ246660	EF560174	JQ246710	JQ246605
Calliphoridae	*Chrysomya chani* Kurahashi, 1979	KT894988	JQ811390	—	KT894969
Calliphoridae	*Chrysomya chloropyga* (Wiedemann, 1818)	JQ246661	JQ246571	JQ246711	JQ246606
Calliphoridae	*Chrysomya marginalis* (Weidemann, 1830)	MH765384	MH716084	—	—
Calliphoridae	*Chrysomya megacephala* (Fabricius, 1794)	JQ246662	EF560175	—	JQ246607
Calliphoridae	*Chrysomya nigripes* Aubertin, 1932	DQ647354	JQ811379	—	—
Calliphoridae	*Chrysomya pinguis* (Walker, 1858)	KY031785	MG597045	—	KT894963
Calliphoridae	*Chrysomya putoria* (Wiedemann, 1830)	JQ246663	EF560176	JQ246712	JQ246608
Calliphoridae	*Chrysomya rufifacies* (Macquart, 1843)	JQ246664	EF560177	JQ246713	JQ24660
Calliphoridae	*Chrysomya thanomthini* Kurahashi & Tumrasvin, 1977	KY020771	—	—	KT894960
Calliphoridae	*Chrysomya villeneuvi* Patton, 1922	KY020756	JX027540	—	KT894975
Calliphoridae	*Cochliomyia aldrichi* Del Ponte, 1938	KX529529	KX529563	—	KX529513
Calliphoridae	*Cochliomyia hominivorax* (Coquerel, 1858)	JQ246665	EF560181	JQ246714	JQ246610
Calliphoridae	*Cochliomyia macellaria* (Fabricius, 1775)	JQ246666	EF560182	JQ246715	JQ246611
Calliphoridae	*Cochliomyia minima* Shannon, 1926	KX529552	KX529596	—	KX529507
Calliphoridae	*Compsomyiops fulvicrura* (Robineau‐Desvoidy, 1830)	FJ025607	—	FJ025428	FJ025504
Calliphoridae	*Compsomyiops verena* (Walker, 1849)	KC568265	KP723296	—	—
Calliphoridae	*Cordylobia anthropophaga* (Blanchard & Berenger‐Feraud, 1872)	JQ246681	JQ246579	JQ246730	JQ246627
Calliphoridae	*Hemilucilia segmentaria* (Fabricius, 1805)	JQ246667	EF560192	JQ246716	JQ246612
Calliphoridae	*Hemilucilia semidiaphana* (Rondani, 1850)	JQ246668	JQ246572	JQ246717	JQ246613
Calliphoridae	*Hemipyrellia fernandica* (Macquart, 1855)	KF839571	—	—	AJ558191
Calliphoridae	*Hemipyrellia ligurriens* (Wiedemann, 1830)	JQ246676	JQ246576	JQ246725	JQ246621
Calliphoridae	*Hemipyrellia pulchra* (Wiedemann, 1830)	KR921680	JQ811336	KC347599	KC538814
Calliphoridae	*Lucilia ampullacea* Villeneuve, 1922	JX295666	JX295789	JX295741	JX295770
Calliphoridae	*Lucilia caesar* (Linnaeus, 1758)	JX295697	JX295798	JX295749	JX295779
Calliphoridae	*Lucilia cluvia* (Walke, 1849)	JN280714	MF097626	—	AJ551440
Calliphoridae	*Lucilia caeruleiviridis* Macquart, 1855	KR752115	MF097633	—	KF839502
Calliphoridae	*Lucilia cuprina* (Wiedemann, 1830)	JQ246677	EF560185	JQ246726	JQ246622
Calliphoridae	*Lucilia eximia* (Wiedemann, 1819)	JQ246678	EF560186	JQ246727	JQ246623
Calliphoridae	*Lucilia fayeae* Whitworth, 2010	JN280720	MF097672	—	KF839510
Calliphoridae	*Lucilia lucigerens* (James, 1971)	KX283408	MF097673	—	—
Calliphoridae	*Lucilia mexicana* Macquart, 1844	JN280725	MF097677	—	AJ551441
Calliphoridae	*Lucilia papuensis* Macquart, 1843	KY031743	JQ811302	—	FR719307
Calliphoridae	*Lucilia porphyrina* Walker, 1856	MF694302	JQ811295	EU661341	MF694310
Calliphoridae	*Lucilia retroversa* (James, 1971)	JN280712	MF097683	—	—
Calliphoridae	*Lucilia rica* Shannon,1926	JN280711	MF097697	—	—
Calliphoridae	*Lucilia richardsi* Collin, 1926	KJ394940	KF825991	—	AJ551442
Calliphoridae	*Lucilia sericata* (Meigen, 1826)	JQ246679	EF560187	JQ246728	JQ246624
Calliphoridae	*Lucilia silvarum* (Meigen, 1826)	MG118579	KF826006	—	FR719316
Calliphoridae	*Pachychoeromyia praegrandis* (Austen, 1910)	JQ246683	JQ246581	JQ246732	JQ246629
Calliphoridae	*Paralucilia fulvinota* (Bigot, 1877)	MH688062	MH699084	MH703525	MH699111
Calliphoridae	*Paralucilia paraensis* (Mello, 1969)	MH987770	MH699119	MH703529	—
Calliphoridae	*Paralucilia pseudolyrcea* (Mello, 1969)	MH987767	MH699087	MH703519	MH699121
Calliphoridae	*Phormia regina* (Meigen, 1826)	JQ246669	EF560190	JQ246718	JQ246614
Calliphoridae	*Protocalliphora azurea* (Fallén, 1817)	FR719180	—	GQ409147	GQ409264
Calliphoridae	*Protocalliphora sialia* Shannon & Dobroscky, 1924	KR672687	—	—	AJ558190
Calliphoridae	*Protophormia terraenovae* (Robineau‐Desvoidy, 1830)	JQ246670	EF560193	JQ246719	JQ246615
Calliphoridae	*Sarconesia chlorogaster* (Wiedemann, 1830)	JQ246674	JQ246574	JQ246723	JQ246619
Calliphoridae	*Sarconesia versicolor* Bigot, 1857	GQ409319	—	GQ409121	_
Sarcophagidae	*Oxysarcodexia thornax* (Walker, 1849)	JQ246695	—	JQ246746	_
Sarcophagidae	*Sarcophaga* (*Neobellieria*) *bullata* Parker, 1916	JQ246696	JQ246593	JQ246748	JQ246644
Sarcophagidae	*Peckia* (*Squamatodes*) *ingens* (Walker, 1849)	—	—	JQ246747	JQ246643

Each marker was aligned with the T‐Coffee online server (Di Tommaso et al. 2011; Notredame et al. 2000) and further concatenated in Mesquite 3.61 (Maddison and Maddison 2019). For the best substitution model and partition scheme, we utilized PartitionFinder 2.1 software (Lanfear et al. 2017). Each molecular marker was treated as a partition, except for COI, which was partitioned based on codon position (1st, 2nd and 3rd positions). The selected partitioning scheme was as follows: GTR + G (COI 1st), GTR + I + G (COI 2nd), F81 + I (COI 3rd), GTR + G (ITS2), GTR + I + G (16S), and GTR + I + G (28S).

The tree was inferred using Bayesian Inference in MrBayes v.3.2.7 (Ronquist et al. 2012), available at the CIPRES Science Gateway v.3.3 (Miller et al. 2015). The models applied to each partition were considered unlinked, employing the following parameters: prset applyto = (all) ratepr = variable, unlink statefreq = (all) revmat = (all) shape = (all) pinvar = (all). Markov chains underwent 10,000,000 generations (nchains = 6; sample frequency = 1000) and a burn‐in of 25% was applied. Posterior probabilities (PP) were assessed for node support in the 50% extended majority‐rule consensus tree.

Lastly, we transformed the tree to ultrametric for comparative analyses with branch lengths proportional to time. We used the “chronos” function (R package Ape (Paradis et al. 2004)), with a relaxed model (lambda = 0) and three calibration points from Cerretti (Cerretti et al. 2017): the divergence between *Calliphora vicina* and *Calliphora vomitoria* , between *Lucilia cuprina* and *Lucilia sericata*, and between *Cochliomyia hominivorax* and *Cochliomyia megacephala*.

### 2.3 Phylogenetics Comparative Analyses

To investigate the tempo and mode of vertebrate parasitism evolution across Calliphoridae, we modeled the rate and direction of change individually for three categorical traits: (i) trophic specialization, (ii) larval food substrate, and (iii) larval developmental temperature. We conducted a discrete multistate trait evolution analysis using BayesTraits v4.0.1 (Pagel and Meade 2006) to estimate ancestral states and transition rates. We used a reversible‐jump Markov Chain Monte Carlo (rjMCMC) approach.

To define priors, we first estimated the transition rates using Maximum Likelihood analysis. We used a uniform hyperprior to determine the range and shape of the gamma prior distribution, with each trait having its own hyperprior specification. Chains were run for 110 million iterations, sampling every 1000 iterations, with the first 10 million discarded as burn‐in.

Using the estimated transition rates, we tested the hypothesis that a direct transition from saprophagous to obligatory parasite is less likely than an indirect pathway involving an intermediate stage (i.e., saprophagous → facultative parasite → obligatory parasite). To evaluate this, we calculated the Bayes Factors (BF) as: BFij = [P(Mi|D)/P(Mj|D)]*[P(Mj)/P(Mi)]. Here, *P*(*Mi|D*) represents the proportion of models where the normalized transition rates from saprophagous to obligatory parasite were lower than the product of the normalized transition rates from saprophagous to facultative parasite and facultative parasite to obligatory parasite. Conversely, *P*(*Mj|D*) represents the proportion of models where this relationship was reversed, meaning the direct transition rate was equal to or greater than the product of the two‐step pathway. *P*(*Mi*) and *P*(*Mj*) represent the probabilities of the hypothesis tested being correct or incorrect due to chance, respectively, and were calculated using Stirling numbers. We performed similar tests for larval food substrate (i.e., “necrotic” → “fresh” or “necrotic” → “both” → “fresh”) and larval developmental temperature (i.e., “variable” → “constant” or “variable” → “both” → “constant”).

Additionally, we tested whether the ancestor was saprophagous, using “necrotic” tissue and “variable” temperature. For simplicity, we describe the analysis only for the saprophagous trait, as the other analyses followed the same methodology. To evaluate this, we also used BF, where here *P*(*Mi|D*) represents the proportion of models where the probability of the ancestor being saprophagous was greater than the combined probability of it being a facultative or obligatory parasite. Conversely, *P*(*Mj|D*) represents the proportion of models where the probability of the ancestor being saprophagous was equal to or lower than the sum of the other two possibilities. *P*(*Mi*) and *P*(*Mj*) here were calculated based on the proportion of the area within a triangle in which the sum of the three ancestral probabilities is equal to 1 and varies uniformly between 0 and 1. BF values between 3 and 12 were considered positive support, and values above 12 were considered strong positive support. Scripts and raw data are available in the Dryad data repository.

## 3 Results

The reconstructed phylogenetic tree was based on a concatenated dataset of 2735 bp (COI = 658 bp; ITS2 = 671 bp; 16S = 441 bp; 28S = 965 bp; Table 2). The tree was fully dichotomous, with Calliphoridae recovered as monophyletic. Overall, the tree was highly supported, with most posterior probabilities above 0.9. The monophyly of all Calliphoridae genera was also recovered, except for the genus *Lucilia*, which was paraphyletic due to the inclusion of *Hemipyrellia*.

**FIGURE 1:**
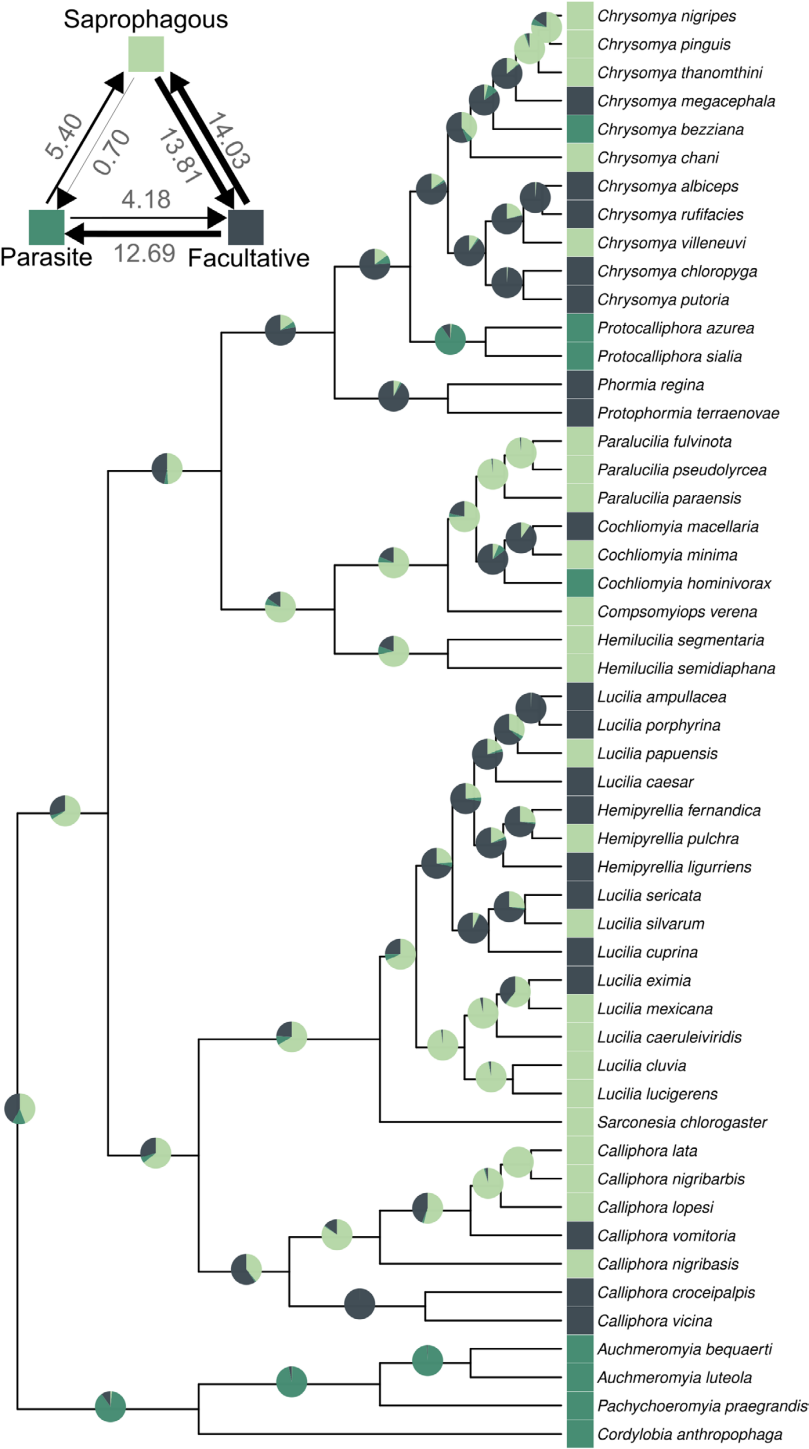
Reconstructed ancestral states and estimated evolutionary transitions of blowfly trophic habits. In each node, pie charts illustrate the estimated probability of the three trophic habits: Obligatory parasite (green), saprophagous (light green) and facultative parasite (dark green). To the top left of the phylogeny estimated transition rates among states are shown.

Our ancestral state estimates for trophic specialization indicated a weak tendency for the ancestral condition to be saprophagous (BF = 1.51). The probability of being saprophagous or a facultative parasite was close (mean estimates 0.44 and 0.42, respectively), followed by a lower probability of a parasitic root (mean estimate 0.14; Figure 1; Data S1). The highest transition rates occurred between saprophagous and facultative parasite habits (Figure 1) and in the transition from facultative to obligatory parasitism (Figure 1). The transition to obligatory parasitism was over 18 times more likely to occur from the facultative parasite habit than from saprophagous (Figure 1). Additionally, our results strongly supported that the transition from saprophagous to obligatory parasite required an intermediate step through the facultative parasite habit (BF = 25.88, Figure 1).

**FIGURE 2:**
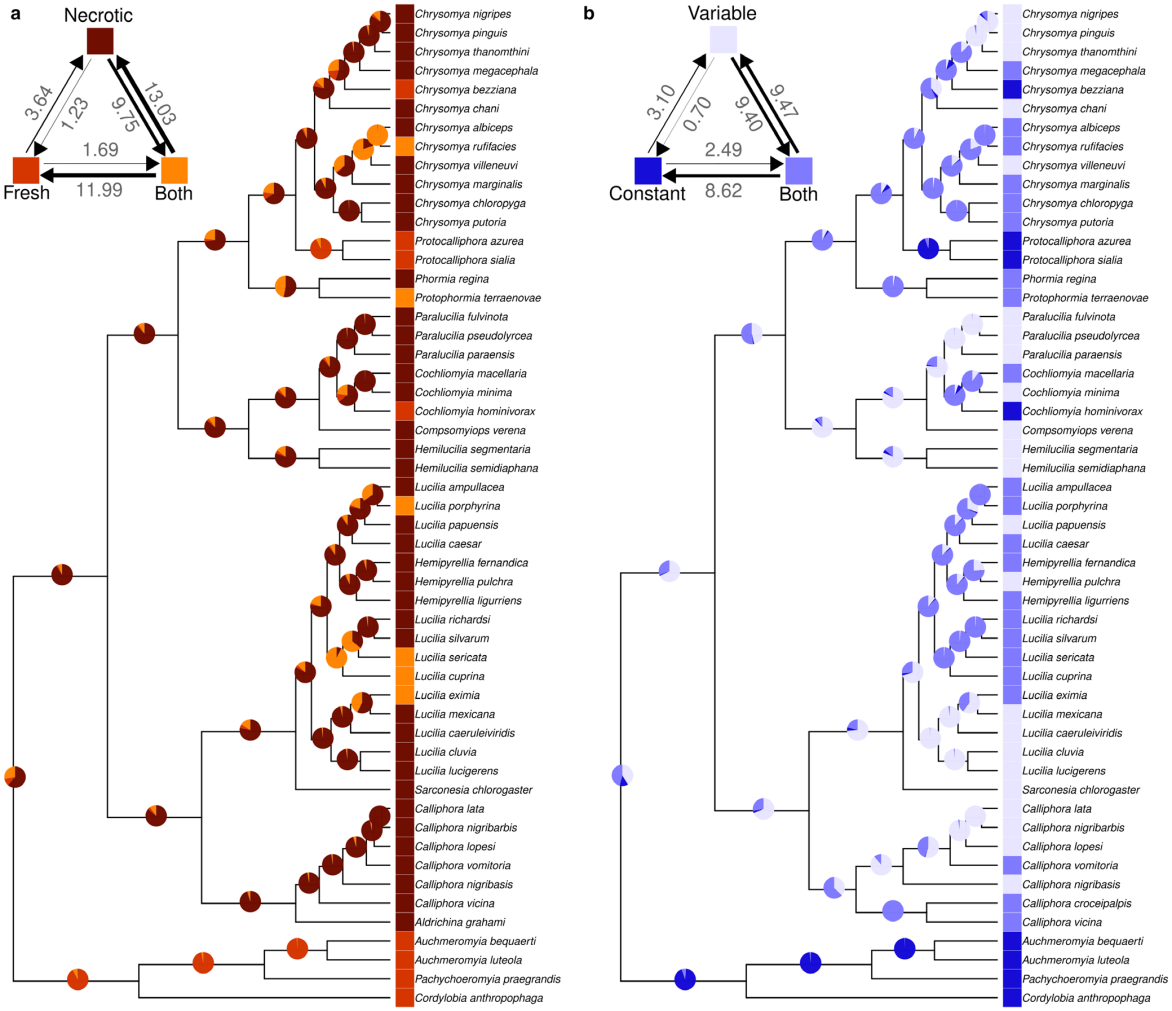
Reconstructed ancestral states and estimated evolutionary transitions for blowfly larval food substrate (a: Shades of red) and developmental temperature (b: Shades of blue). Pie charts plotted in each node represent the estimated probability of the larval food substrate (a: Fresh, necrotic, and both) and developmental temperature (b: Constant, variable, and both). The top left of each phylogeny presents estimated transition rates among states.

Our ancestral estimate analysis for larval food substrates indicated a higher probability of the ancestral condition being “necrotic” tissue (BF = 8.53; mean estimate 0.61), in contrast to “fresh” tissue and “both” types (mean estimates 0.11 and 0.28, respectively; Figure 2a; Data S1). The highest transition rates were from “both” (when the species has access to either necrotic or fresh tissue) to “necrotic” and from “both” to “fresh” (Figure 2a). The use of “fresh” tissue as larval food was the least common, observed in only 8 out of 53 species, and was more likely to transition from a condition with access to “both” types than from “necrotic” tissue (Figure 2a). The transition to the use of “fresh” tissue was over nine times more likely to occur from the use of “both” than from “necrotic” tissue (Figure 2a). This analysis also supported that the transition from the use of “necrotic” to “fresh” tissue required the use of “both” types of substrates as an intermediate step (BF = 3.10; Figure 2a).

Ancestral state estimates for larval developmental temperature revealed uncertainty regarding whether the ancestral condition was “variable” or not (BF = 0.75). The probability of being “variable” or “both” (mean estimates 0.41 and 0.46, respectively) was followed by a lower probability of a “constant” temperature (mean estimate 0.13; Figure 2b; Data S1). The highest transition rates occurred between “both” (species with access to either variable or constant temperature) and “variable” and in the transitions from “both” to “constant” temperature (Figure 2b). The transition to the use of “constant” temperature was over 12 times more likely to occur from the use of “both” than from “variable” temperature (Figure 2b). Our analysis strongly supported that the transition from the use of “variable” to “constant” temperature for larval development required access to “both” temperatures as an intermediated step (BF = 16.68; Figure 2b).

## 4 Discussion

In this study, we aimed to unravel the evolutionary steps leading to vertebrate parasitism in blowflies. By reconstructing a phylogeny, we revealed a fully dichotomous tree with well‐supported monophyly of Calliphoridae, laying the groundwork for exploring trophic specialization, larval food substrate, and developmental temperature across the blowfly phylogeny.

While previous studies (Stevens 2003; Stevens and Wallman 2006; McDonagh and Stevens 2011; Nasser et al. 2021) have explored the phylogeny of Calliphoridae and discussed the origin and evolution of myiasis, they primarily adopted a descriptive approach. These studies often relied on evaluating terminal taxa on a phylogenetic tree to infer the origin of myiasis, lacking a formal statistical framework to test the evolutionary trajectory of this specialized trait. In contrast, our study is the first to explicitly investigate the evolution of myiasis by formally testing the evolutionary trajectory of myiasis using a Bayesian phylogenetic approach to reconstruct ancestral states and model the transitions between states. Our contribution lies in providing a formal statistical evaluation of myiasis evolution, which was not a primary focus of previous studies. Additionally, while some studies (McDonagh and Stevens 2011; Nasser et al. 2021) employed a broader taxon sampling, we deliberately selected taxa that are particularly relevant to the specific evolutionary transitions between saprophagy and parasitism within myiasis. This focused approach allows us to optimize our study design for answering targeted evolutionary questions and ensures sufficient statistical power to infer ancestral states.

Our analysis of ancestral estimates for the trophic specialization slightly favors the ancestor of Calliphoridae being saprophagous, although this result does not strongly support the hypothesis tested. It is most likely that the ancestor state was either a saprophagous or facultative parasite. Similarly, the ancestral developmental temperature condition was likely “variable” or “both” types (variable and constant temperature). This finding suggests adaptability to diverse developmental environments, such as corpses or hosts with distinct thermoregulatory strategies (heterothermic or homeothermic), which are conditions used by saprophagous or facultative species. Additionally, our ancestral estimate for larval food substrates pointed to “necrotic” tissue. While necrotic tissue is typically associated with saprophagous species, it can also be exploited by facultative parasites. These findings align with the proposed origin by Zumpt (1965). According to this hypothesis, parasitism could have originated from saprophagous larvae, exploring dead tissues and eventually changing to explore living tissues on a living host, which might have been either homeothermic or heterothermic.

While our analysis of trophic specialization expands our perspective into the evolution of myiasis in blowflies, it is important to note that this classification sometimes is used in the literature primarily focusing on breeding environment and female oviposition behavior. However, the complete parasitic lifestyle involves a range of adaptations, particularly in larval physiology and behavior, which are not fully discussed in the literature. For instance, facultative parasites may lay eggs on living hosts, but their larvae may feed on necrotic tissue, fresh tissue, or a combination of both. A more comprehensive understanding of the evolution of myiasis requires considering the specific feeding habits of larvae. Therefore, it was important to also analyze the larval food substrate and developmental temperature traits. Unfortunately, detailed information on larval feeding habits is often lacking for many blowfly species, especially in natural conditions. There are some studies describing larval survival in laboratory conditions and/or on artificial media, but these environments may not accurately reflect the natural ecological roles of the species; thus, we did not consider laboratory studies in our analyses. The lack of information on larval feeding habits highlights the need for future studies to investigate the natural ecological preferences of larval stages. By incorporating a more detailed approach to classifying blowfly ecology, including specific information about female and larval behavior and physiology, as we did in this study, further studies can generate and test new hypotheses regarding the evolutionary pathways leading to parasitism in blowflies in various ecological contexts, including other blowfly taxa that parasitize other organisms like woodlice, earthworms, and termites.

Our analyses also highlighted the pivotal role of facultative parasitism as an intermediate step for the evolution of myiasis in blowflies. Facultative parasitism emerged as a crucial precondition for obligate parasitism in the estimated transitions of the trophic specialization analysis. Furthermore, the larval food substrate analysis suggests that the “fresh” tissue (exclusively used by obligatory parasites) evolved from the use of “both” types of tissues (necrotic and fresh tissue). This indicates an intermediate step for the evolution of the parasitic larval food substrate. The same was observed for the developmental temperature. The “constant” temperature (exclusively used by obligatory parasites) most likely evolved from an intermediate stage involving the use of “both” types of temperature conditions (variable and constant). This intermediate condition could represent a cadaver, heterothermic, or homeothermic host and is experienced by the facultative parasites. These analyses confirm the continuous path for parasitism evolution proposed by Zumpt (Zumpt 1965), originating from a saprophagous ancestor and passing through a facultative state. Moreover, pre‐adaptations to facultative parasitism (e.g., morphological, behavioral, physiological and genetic) highlight the importance of certain ecological conditions (e.g., host availability and competition) that can trigger the transition from a free‐living to a parasitic lifestyle (Luong and Mathot 2019). These conditions favoring the evolution of facultative parasitism have been observed in a range of taxonomic groups (Viney 1997; Luong et al. 2017; Houck and Cohen 1995; Washburn et al. 1988; Morin et al. 1993; Stefanik et al. 2014; Gomes et al. 2018). This concept aligns with our findings, emphasizing the significance of facultative parasitism as a transitional stage in the evolution of vertebrate parasitism in blowflies.

Explanations based on pre‐adaptations elucidate the processes of evolutionary changes in lineages transitioning from free‐living to facultative parasitism. However, the mechanisms underlying this evolutionary transition, including the molecular, physiological, and behavioral processes involved, remain unclear. This is particularly important in pest species like blowflies, as understanding the biological mechanisms underlying vertebrate parasitism (myiasis) transitions can offer new research opportunities for enhancing pest control methods.

In conclusion, our study contributes to the discussion of the complex evolutionary history of blowfly vertebrate parasitism, providing a detailed perspective on the origin and transitions towards myiasis. Future research can further explore the mechanisms driving these shifts and the implications of facultative parasitism in shaping the diverse adaptations seen in the vertebrate parasitic lifestyle. Additionally, our findings into myiasis evolution could serve as a foundation for future studies on the origins and transitions of parasitism in several ecological contexts, including other taxa.

## Author Contributions

**Gisele Antoniazzi Cardoso:** conceptualization (equal), data curation (equal), investigation (equal), methodology (equal), project administration (supporting), validation (equal), visualization (equal), writing – original draft (equal), writing – review and editing (equal). **Vanessa A. S. Cunha:** conceptualization (equal), data curation (equal), investigation (equal), methodology (equal), project administration (supporting), validation (equal), visualization (equal), writing – original draft (equal), writing – review and editing (equal). **Bruno C. Genevcius:** conceptualization (equal), data curation (equal), formal analysis (equal), investigation (supporting), project administration (equal), software (equal), validation (equal), visualization (equal), writing – original draft (equal), writing – review and editing (equal). **Tais Madeira‐Ott:** data curation (equal), formal analysis (equal), investigation (equal), methodology (equal), software (equal), writing – original draft (supporting), writing – review and editing (supporting). **Bárbara Maria de Andrade Costa:** methodology (equal), writing – original draft (supporting), writing – review and editing (supporting). **Daniela Munhoz Rossoni:** methodology (equal), writing – original draft (supporting), writing – review and editing (supporting). **Patricia Jacqueline Thyssen:** resources (supporting), supervision (equal), writing – original draft (supporting), writing – review and editing (supporting). **Tatiana Teixeira Torres:** conceptualization (equal), funding acquisition (lead), project administration (lead), resources (equal), supervision (lead), validation (equal), writing – original draft (equal), writing – review and editing (equal).

## Conflicts of Interest

The authors declare no conflicts of interest.

## Supporting information


Data S1.

## Data Availability

All data supporting this study are publicly available in the Dryad digital repository under the DOI 10.5061/dryad.nzs7h4522.
